# HCAR Is a Limitation Factor for Chlorophyll Cycle and Chlorophyll *b* Degradation in Chlorophyll-*b*-Overproducing Plants

**DOI:** 10.3390/biom10121639

**Published:** 2020-12-05

**Authors:** Xuan Zhao, Ting Jia, Xueyun Hu

**Affiliations:** 1 Key Laboratory of Plant Functional Genomics of the Ministry of Education, Yangzhou University, Yangzhou 225009, China; mx120190786@yzu.edu.cn (X.Z.); tingj2012@yzu.edu.cn (T.J.); 2 Joint International Research Laboratory of Agriculture and Agri-Product Safety of the Ministry of Education of China, Yangzhou University, Yangzhou 225009, China; 3 College of Bioscience and Biotechnology, Yangzhou University, Yangzhou 225009, China

**Keywords:** chlorophyll cycle, chlorophyll degradation, HCAR, leaf senescence, cell death

## Abstract

The chlorophyll (Chl) cycle is the metabolic pathway for Chl *a* and Chl *b* inter-conversion. In this pathway, Chl *b* is synthesized from Chl *a* by the catalyzing action of chlorophyllide *a* oxygenase (CAO). In contrast, Chl *b* is firstly reduced to produce 7-hydroxymethyl Chl (HMChl) *a*, which is catalyzed by two isozymes of Chl *b* reductase (CBR), non-yellow coloring 1 (NYC1) and NYC1-like (NOL). Subsequently, HMChl *a* is reduced to Chl *a* by HMChl *a* reductase (HCAR). CAO plays a pivotal role in Chl *a*/*b* ratio regulation and plants over-accumulate Chl *b* in CAO-overexpressing plants. NYC1 is more accumulated in Chl-*b*-overproducing plants, while HCAR is not changed. To investigate the role of HCAR in Chl cycle regulation, the Chl metabolites of Chl-*b*-overproducing plants were analyzed. The results showed that HMChl *a* accumulated in these plants, and it decreased and the Chl *a*/*b* ratio increased by overexpressing HCAR, implying HCAR is insufficient for Chl cycle in Chl-*b*-overproducing plants. Furthermore, during dark-induced senescence, the non-programmed cell death symptoms (leaves dehydrated with green color retained) of Chl-*b*-overproducing plants were obviously alleviated, and the content of HM pheophorbide (HMPheide) *a* and Pheide *b* were sharply decreased by overexpressing HCAR. These results imply that HCAR is also insufficient for Chl degradation in Chl-*b*-overproducing plants during senescence, thus causing the accumulation of Chl metabolites and non-programmed cell death of leaves. With these results taken together, we conclude that HCAR is not well regulated and it is a limiting factor for Chl cycle and Chl *b* degradation in Chl-*b*-overproducing plants.

## 1. Introduction

Chlorophyll (Chl) is the key pigment responsible for harvesting solar energy and producing charge separation and electron transport during photosynthesis in green plants. Once it is synthesized during greening, Chl must be incorporated into its target apo-proteins to build functional photosynthetic proteins [1]. In contrast, Chl should be degraded in coordination with its apo-proteins during senescence. This is because free Chl can produce reactive oxygen species, causing cell death [2]. Additionally, intermediate molecules of Chl metabolism can also be toxic to plant cells. For example, the accumulation of pheophorbide *a* (Pheide *a*) induces non-programmed cell death (non-PCD), shown by leaves dehydrated but with green color retained in both light-dependent and light-independent manners [3,4,5]. Therefore, to avoid non-PCD occurring, Chl metabolism is finely regulated by genetic and environmental conditions throughout all the developmental stages of plants [6,7,8].

**Figure 1:**
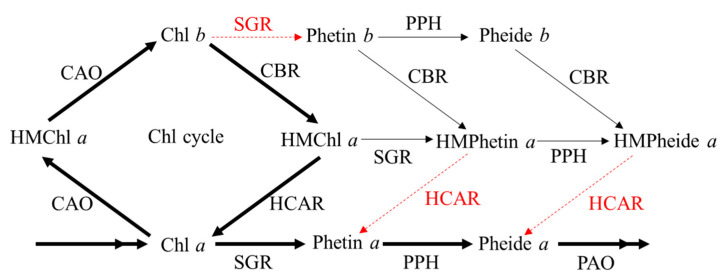
Chlorophyll cycle and degradation pathway in land plants. Thick arrows represent the major Chl metabolic pathways. Thin arrows represent the possible minor Chl metabolic pathways. Dotted arrows and the names of the enzymes beside them are in red, which indicate that the labelled enzymes have no activity to catalyze the corresponding steps in vitro. Phetin: pheophytin.

Green plants contain two Chl species, Chl *a* and Chl *b*, with different absorption spectra. The only different structure between Chl *a* and Chl *b* is the side chain at C-7, which is a methyl group in Chl *a* and a formyl group in Chl *b*. Chl *a* is located in both the inner and peripheral antennae, and it is essential for photochemistry, while Chl *b* is found in peripheral antenna complexes (also named light-harvesting complexes (LHC), LHCI and LHCII), and it is necessary for stabilizing the major LHC [9]. Chl *a* and Chl *b* can interconvert to each other in the Chl cycle (Figure 1) [10,11]. Chl *b* is synthesized from Chl *a* by the catalyzing action of a unique enzyme, chlorophyllide *a* oxygenase (CAO) [12,13], which converts Chl *a* to Chl *b* via 7-hydroxymethyl Chl *a* (HMChl *a*). Chl *b* is capable of being reconverted to Chl *a* by two reduction steps. First, the formyl group of Chl *b* is reduced to produce HMChl *a*, which is catalyzed by two isozymes of Chl *b* reductase (CBR), non-yellow coloring 1 (NYC1) and NYC1-like (NOL) [14]. Second, HMChl *a* is reduced to Chl *a* by HMChl *a* reductase (HCAR) [15]. The Chl cycle is required to finely regulate the Chl *a*/*b* ratio, which is important for acclimation of plants to the light environment. For example, if light is too weak for plants, CAO must be upregulated to catalyze the fittest amount of Chl *a* to Chl *b* in order to construct more LHCs for harvesting more light. In contrast, if the light intensity is too strong for plants, excess Chl *b* must be degraded to Chl *a* to destruct the excess LHCs to avoid photodamage. On the other hand, the degradation of Chl *b* also requires the conversion of Chl *b* to Chl *a* during leaf senescence, because magnesium (Mg) cannot be extracted from Chl *b* by Mg-dechelatase (SGR), which catalyzes the first step of Chl *a* degradation [16]. Meanwhile, another Chl degradation enzyme, Pheide *a* oxygenase (PAO), can only catalyze the opening of the porphyrin ring of Pheide *a*, which is the product catalyzed from Chl *a* by SGR and pheophytin pheophorbide hydrolase (PPH) [17].

Various mechanisms cooperatively and finely regulate the Chl cycle. Several lines of evidence have shown that Chl *a*/*b* ratio seems to be determined by the Chl *a* to *b* conversion in the Chl cycle. Transgenic *Arabidopsis*, in which the content of CAO protein is elevated, possesses a higher level of Chl *b* and a lower level of Chl *a*, resulting in a much lower Chl *a*/*b* ratio, compared with wild-type plants [18]. In contrast, plants lacking CBR or HCAR show similar Chl *a*/*b* ratios with wild-type plants during their vegetative phase [15,19]. The activity of CAO is majorly regulated on the post-transcriptional level, while transcriptional control only plays a minor role in the regulation of CAO activity [20,21,22]. In *Arabidopsis*, mature CAO contains three domains: the N-terminal domain (also termed the A domain), middle domain (also termed the B domain) and C-terminal domain (also termed the C domain). The stability of CAO is regulated by Clp protease, which recognizes the degron sequence in the A domain and may drag CAO into its proteolytic domain to complete the degradation of CAO [23,24,25]. The degron sequence of CAO-attached green fluorescent protein (GFP) was destabilized in the chloroplasts of both wild-type *Arabidopsis* and the CAO-deficient mutant, while the whole A domain-attached GFP was destabilized in wild-type but stabilized in the CAO-deficient mutant [26]. These results suggested that the destabilization of CAO is Chl-*b*-dependent, and not only the degron of the A domain is necessary for this process [9].

The regulation of the degradation of Chl *b* to Chl *a* in the Chl cycle was also investigated. It was found that both *NYC1* mRNA and the NYC1 protein were increased in response to dark-induced senescence, while they were in low abundance in green leaves [27]. In contrast, the transcription levels of *NOL* and *HCAR* were sharply increased during the greening of etiolated seedlings, suggesting that NOL and HCAR may play major roles in Chl turnover during the vegetative stage [28]. On the post-transcription level, it was found that the amount of NYC1 was greatly increased in plants that overexpressed CAO without the A domain, while the transcription of *NYC1* was not obviously changed compared with the wild-type [27]. It was demonstrated that energetically uncoupled LHC with Chl *b* is related to the NYC1 level, suggesting a feedforward regulation of NYC1 by the energetically uncoupled LHC [27]. Although the truncated CAO (only BC domain) and NYC1 amounts were increased in CAO over-accumulating plants, their HCAR amount was similar to the wild-type both before and after dark-induced senescence [27]. Plants lacking HCAR not only accumulate HMChl *a* but also accumulate Pheide *a*, resulting in non-PCD during dark-induced senescence [15]. It is hypothesized that in order to avoid the non-PCD induced by the accumulation of Pheide *a*, HCAR must immediately convert HMChl *a* to Chl *a* [29]. Therefore, HCAR activity should be high enough to meet the requirement of Chl *b* to Chl *a* conversion in these plants, in which the increase of HCAR abundance is unnecessary.

In this study, we investigated the role of HCAR in the Chl cycle by analyzing plants that were overexpressing *CAO* (without A domain), and the results implied that HCAR activity is insufficient for catalyzing HMChl *a* reduction in Chl-*b*-overproducing plants. When HCAR was overexpressed in these Chl-*b*-excess plants, the Chl *b* level was reduced, and the Chl *a*/*b* ratio was increased from approximately 0.8–1 to 1.6–2, indicating that HCAR is one of the limiting enzymes for Chl *b* turnover when Chl *b* is overproduced. During natural senescence and dark-induced senescence, overexpressing HCAR alleviated the non-PCD symptoms of Chl *b*-over-accumulating plants. Moreover, HMPheide *a* and Pheide *b* were decreased, and Chl *b* degradation was accelerated. Taken together, we conclude that HCAR is a limiting factor for the Chl cycle and Chl *b* degradation in Chl-*b*-overproducing plants.

## 2. Materials and Methods

### 2.1. Plant Material and Growth Conditions

*Arabidopsis* PhCAO (*Prochlirothrix hollandica* CAO-overexpressing) and BCG (the B and C domain of CAO fused with GFP-overexpression) plants were generated previously [18,25]. The plants were grown in soil under long-day conditions (16 h light/8 h dark) with fluorescent light at 80–100 μmol photons m^−2^ s^−1^ at 23 °C. To analyze Chl degradation during dark-induced senescence, 4-week-old *Arabidopsis* plants were incubated in darkness for 6 days.

### 2.2. Construction and Arabidopsis Transformation

The full-length complementary DNA (cDNA) of *Arabidopsis HCAR* (At1g04620) gene was amplified and introduced into the Gateway entry vector pENTR4-Dual and then introduced into the Gateway-compatible binary vector pEarleyGate100 by LR reaction [30]. The construct was introduced into Agrobacterium tumefaciens (strain GV3101) and subsequently transformed into *Arabidopsis* using a floral dip method [31]. HCAR-overexpressing transformants were screened by spraying with the herbicide glufosinate ammonium (Basta).

### 2.3. Pigment Preparation and Chl Analysis

First, leaves were harvested and weighed. Second, Chl and its metabolic intermediate molecules were extracted from leaf tissue by homogenization with pre-cooled acetone [32]. The extracts were subsequently centrifuged for 5 min at 20,000× *g* and 4 °C, and the supernatant was analyzed by HPLC using a symmetry C8 column (150 mm in length, 4.6 mm in I.D.; Waters, Milford, MA, USA) according to the method of Zapata et al. [33]. Pigment concentrations were estimated from the absorption monitored at 410 nm. Standard Chl *a* and Chl *b* were purchased from Juntec Co. Ltd., Odawara, Japan, while Pheide *a* was purchased from Wako Pure Chemical Industries, Ltd. Japan.

### 2.4. Chl Fluorescence Measurements

The method used for Chl fluorescence measurement was similar to a previous description [27]. The maximal photochemical efficiency of photosystem II (Fv/Fm) was measured using a PAM-2000 fluorometer (Walz,** Effeltrich, Germany) after the plants were adapted to darkness for 15 min at room temperature.

## 3. Results

### 3.1. HMChl a was Accumulated in Chl-b-Overproducing Plants

**Figure 2:**
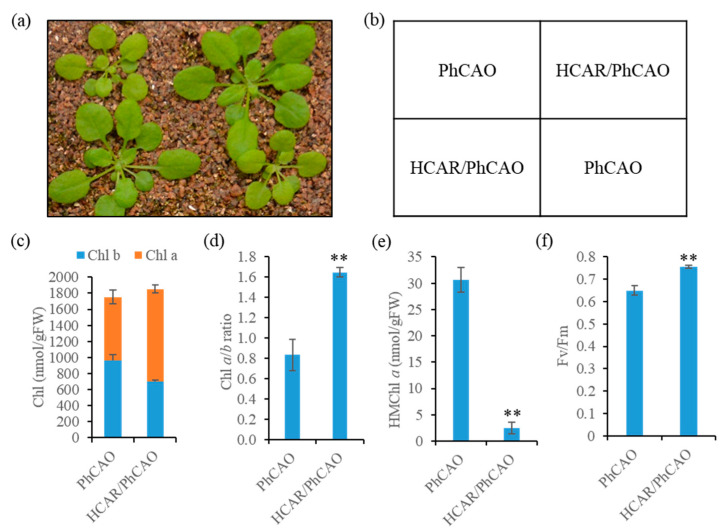
The changes of growth, Chl (chlorophyll) and Chl fluorescence parameters upon overexpressing HCAR (7-hydroxymethyl Chl *a* (HMChl *a*) reductase) in PhCAO (*Prochlirothrix hollandica* chlorophyllide *a* oxygenase-overexpressing). (**a**) PhCAO and HCAR/PhCAO plants and (**b**) their location map. (**c**) Chl *a* and *b* contents, (**d**) Chl *a*/*b* ratios, (**e**) HMChl *a* contents and (**f**) Fv/Fm ratios of PhCAO and HCAR/PhCAO plants. Values are means ± SD of three independent experiments. Asterisks indicate significant difference compared to PhCAO (Student’s *t*-test, ** *p* < 0.01).

To examine whether HCAR can catalyze the reduction of HMChl *a* immediately when CBR is induced, Chl and HMChl *a* content in Chl-*b*-overproducing plants was measured by HPLC analysis (Figure 2). As was reported, Chl *a*/*b* ratio in PhCAO plants was approximately 0.8 (Figure 2d) [18], and the Chl *a*/*b* ratio in BCG plants was approximately 1.0 (Supplementary Materials Figure S1b) [25]. In addition, we found that HMChl *a* accumulated in amounts of approximately 30 nmol and 65 nmol per gram of fresh weight of leaves in phCAO and BCG plants, respectively (Figure 2e and Supplementary Materials Figure S1c), indicating that HCAR activity is insufficient to immediately catalyze the reduction of HMChl *a* to Chl *a* in these plants.

### 3.2. Chl b and HMChl a Was Decreased by Overexpressing HCAR in Chl b-Over-Accumulating Plants

To further demonstrate whether the accumulation of HMChl *a* in Chl *b*-over-accumulating plants is the result of insufficient HCAR activity, HCAR was overexpressed in PhCAO and BCG, respectively. Hereafter, the HCAR-overexpressing plants are referred to as HCAR/PhCAO plants and HCAR/BCG plants, respectively. HCAR/PhCAO plants showed similar Chl contents to those of PhCAO plants; however, the amount of Chl *b* was decreased and that of Chl *a* was increased, resulting in Chl *a*/*b* ratio changing from approximately 0.8 to 1.6 (Figure 2c,d). The content of HMChl *a* was also sharply decreased in HCAR/PhCAO plants (Figure 2e). Under our growth conditions, the Fv/Fm ratio of PhCAO plants was approximately 0.6, while it recovered to approximately 0.75 by overexpressing HCAR, which was very close the Fv/Fm value of wild-type (approximately 0.79) (Figure 2f) [34]. These results were consistent with the finding that HCAR/PhCAO plants grow faster compared to PhCAO plants (Figure 2a). Similar results were acquired by comparing the HCAR/BCG plants with BCG plants (Supplementary Materials Figure S1).

### 3.3. Non-PCD Was Alleviated by Overexpressing HCAR in Chl b-Over-Accumulating Plants during both Natural and Dark-Induced Senescence

**Figure 3:**
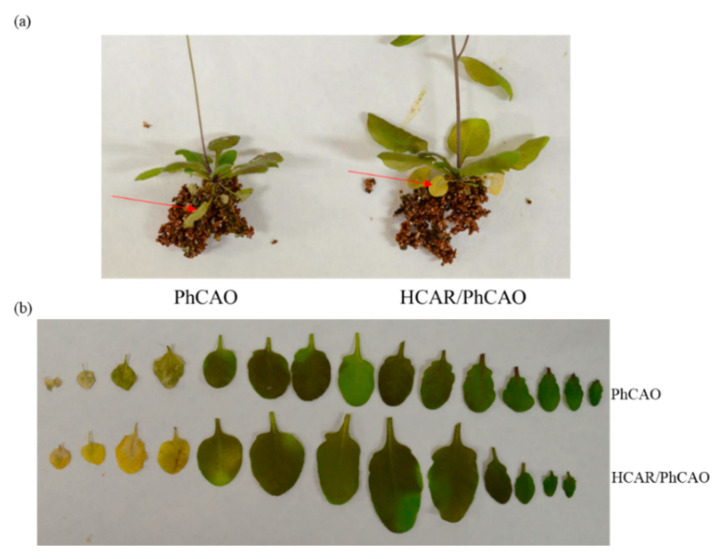
Overexpressing HCAR alleviates the non-PCD (non-programmed cell death) symptoms in PhCAO during natural senescence. Plants were grown under long-day growth conditions for 7 weeks, and (**a**) the whole plants and (**b**) their all detached leaves were imaged with a digital camera. Red arrows indicate the senescence symptoms of No. 3 or 4 leaves of plants.

**Figure 4:**
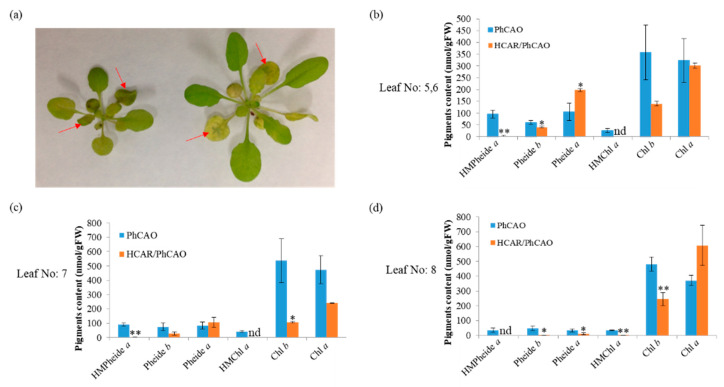
Analysis of the senescence phenotype and the accumulation of Chl and its degradation intermediate molecules after dark-induced senescence. For this, 4-week-old PhCAO and HCAR/PhCAO plants were incubated in darkness for 6 days. (**a**) Overexpressing HCAR alleviates the non-PCD symptoms in PhCAO during dark-induced senescence. Red arrows indicate the senescence symptoms of No. 5 and 6 leaves of plants, which were put in darkness for 6 days. (**b**–**d**) The pigment contents of dark-induced senescent leaves 5-6, 7 and 8, respectively. Pigment measurements were performed on three biological replicates. Error bars indicate the SDs of the biological replicates. Asterisks indicate significant difference compared to PhCAO (Student’s *t*-test, * *p* < 0.05, ** *p* < 0.01). nd indicates not detected.

During leaf senescence, Chl will be degraded, resulting in leaf yellowing. To test the role of HCAR in Chl degradation during leaf senescence, we first observed the natural senescence phenotypes of PhCAO and HCAR/PhCAO plants. Non-PCD heavily occurred in the No. 3 and 4 leaves (the leaf numbers were counted from the oldest to youngest of plant) of 7-week-old PhCAO plants (Figure 3 and Figure S2). These leaves became dehydrated but retained their green color. In contrast, the leaves in the same position of HCAR/PhCAO plants were yellow. These results indicate that overexpressing HCAR alleviated the non-PCD symptoms and promoted Chl degradation in senescent leaves of PhCAO plants. Subsequently, 4-week-old plants were incubated in darkness for 6 days to induce senescence. After incubation, the old leaves, particularly the No. 5 and 6 leaves of PhCAO plants, became dehydrated with green color retained (Figure 4), while most parts of the No. 5 and 6 leaves of HCAR/PhCAO plants were yellow, with only the area near the main vein showing green color. The Chl metabolic intermediates in the leaves of these dark-treated plants were also analyzed. The results showed that the No. 8 leaves of both lines retained similar amounts of Chl, while the No. 5–6 and No. 7 leaves of HCAR/PhCAO plants retained much less Chl than that of PhCAO plants (Figure 4). In addition, the Chl *b* content in the leaves of HCAR/PhCAO plants was obviously less than that of PhCAO plants. The results showed that HMChl *a* was accumulated in all the senescent leaves of PhCAO plants yet rarely accumulated in HCAR/PhCAO plants. Consistent results were also obtained by measuring HMPheide *a* content, the metabolic products of HMChl *a* by SGR, and PPH. All these results suggest that the degradation of Chl *b* and HMChl *a* becomes more unobstructed during leaf senescence due to overexpressing HCAR in PhCAO plants. Surprisingly, we found that Pheide *a* was accumulated in the No. 5–6 and No. 7 leaves of HCAR/PhCAO plants, and the amount was no less than in the same-numbered leaves of PhCAO plants. In contrast, HMPheide *a* and Pheide *b* were decreased. These results indicate that the amount of Pheide *a* is not consistent with the severity of non-PCD symptoms. It is possible that the accumulation of HMPheide *a* and Pheide *b* was also related to the non-PCD symptoms.

## 4. Discussion

HCAR is a unique enzyme that catalyzes HMChl *a* to Chl *a* in the Chl cycle [15]. In wild-type plants, HCAR usually catalyzes the reduction of HMChl *a* immediately, and therefore, HMChl *a* content is under-detectable. However, knowledge about the role of HCAR in Chl *b* turnover and the degradation pathway is largely unreported. In this study, by analyzing Chl-*b*-overproducing plants and the lines that overexpress HCAR in Chl-*b*-overproducing plants, the results demonstrated that HMChl *a* accumulated in Chl-*b*-overproducing plants, while overexpressing HCAR in these plants decreased the HMChl *a* and Chl *b* levels and alleviated the non-PCD symptoms during leaf senescence.

### 4.1. HCAR Activity Is Insufficient for Degrading HMChl a Immediately in PhCAO and BCG Plants

PhCAO and BCG are both CAO-overexpressing transgenic plants that produce ultra-high Chl *b* levels. In previous studies, it was found that NYC1 is accumulated in these plants because energetically uncoupled LHCs with Chl *b* feedforward to trigger the accumulation of NYC1 levels [27]. In contrast, the HCAR level in PhCAO and BCG plants is similar to that in wild-type plants, which implies that excess Chl *b* cannot regulate HCAR. There are two contradictory possibilities regarding this result. One possibility is that the catalytic activity of HCAR is very high, and the amount of HCAR is sufficient to degrade the HMChl *a* produced from excess Chl *b* immediately; therefore, upregulation of HCAR is not required. Another possibility is that the catalytic activity of HCAR is not well regulated; thus, HCAR activity is insufficient for degrading HMChl *a* immediately in Chl-*b*-overproducing plants. Our results clearly showed that both PhCAO and BCG plants accumulate HMChl *a* not only during the vegetative stage but also during natural and dark-induced senescence (Figure 2, Figure 4 and Figure S1). Furthermore, when HCAR was overexpressed in PhCAO and BCG plants, respectively, HMChl *a* and Chl *b* decreased sharply (Figure 2, Figure 4 and Figure S1). These results demonstrate that HCAR is a limiting enzyme in the backward reaction (Chl *b* to Chl *a*) of the Chl cycle in Chl-*b*-overproducing plants. It is still unclear why the enzyme NYC1 in the first reduction step is upregulated, while the unique enzyme HCAR in the second reduction step is not accordingly upregulated. It is possible that too much Chl *b* accumulation is more dangerous to plants than the accumulation of an acceptable level of HMChl *a*.

### 4.2. Accumulation of HMChl a Feedback Affects the Degradation of Chl b

Interestingly, upon overexpressing HCAR in PhCAO and BCG plants, not only the content of HMChl *a* decreased, but also the level of Chl *b* decreased, and more than 20% of Chl *b* was converted to Chl *a* (Figure 2 and Figure S1), resulting in an obvious increase of the Chl *a*/*b* ratio. These results suggest the possibility that the accumulation of HMChl *a* has a feedback effect on the reduction of Chl *b* by CBR. This possibility is also supported by the results that HCAR lacking mutant only accumulates HMChl *a* at low levels (approximately 2% of the total Chl) [15], and the Chl *b* degradation rate of a rice HCAR-lacking mutant is much slower than that of wild-type during dark-induced leaf senescence [35].

### 4.3. Pheide a Is Not the Only Chl Metabolite that Causes Non-PCD in Chl-b-Overproducing Plants

In the leaves of PhCAO and BCG plants, cell death with green color occurred during both natural senescence and dark-induced senescence. During natural senescence, the old leaves of Chl-*b*-overproducing plants showed clear non-PCD symptoms. One hypothesis is that the excessive HMChl *a* enters the binding sites of Chl in photosystem proteins, leading to severe non-PCD symptoms [35]. However, this idea is not consistent with the young green leaves of PhCAO and BCG plants also being able to accumulate HMChl *a*, while they never show clear non-PCD symptoms. In addition, the leaves of *hcar* mutant also accumulate HMChl *a* without non-PCD symptoms before dark treatment [15,35]. Another hypothesis is that the activity of PAO is inhibited via HMChl *a*; thus, Pheide *a* is accumulated during leaf senescence, specifically causing non-PCD in senescent leaves [15]. However, we found that even more amounts of Pheide *a* were accumulated in HCAR/PhCAO than in PhCAO plants (Figure 4). In contrast, HMPheide *a* and Pheide *b* were accumulated in PhCAO plants, but not or much less accumulated in HCAR/PhCAO plants during dark-induced leaf senescence. It is possible that the accumulation of HMPheide *a*, Pheide *b* and Pheide *a* in total caused the more severe non-PCD symptoms in PhCAO than in HCAR/PhCAO plants.

## 5. Conclusions

Our results demonstrate that HCAR is a limiting factor for the Chl cycle and Chl *b* degradation in Chl-*b*-overproducing plants. Plants do not possess a regulation mechanism for HCAR that can respond to the excess of Chl *b* and HMChl *a*, and degrade HMChl *a* immediately. In Chl-*b*-overproducing plants, the expression of HCAR is not enhanced with the increase of Chl *b* and NYC1, and its activity is insufficient for Chl *b* turnover and degradation, resulting in the accumulation of HMChl *a* and ultra-high content of Chl *b*. During senescence, the accumulation of HMPheide *a* and Pheide *b* in Chl-*b*-overproducing plants is also partly because of the insufficient HCAR activity for Chl *b* degradation, and that they may function as toxic molecules causing leaf non-PCD. It was reported that HMChl *a* was accumulated in wild-type under some conditions [15], implying HCAR activity was limited in those cases. Future work should address the conditions under which HMChl *a* stably accumulates in plants, and whether overexpressing HCAR can help plants overcome the stresses that are produced in these conditions.

## Supplementary Materials

The following are available online at https://www.mdpi.com/2218-273X/10/12/1639/s1. Figure S1: The changes of Chl and Chl fluorescence parameters upon overexpressing HCAR in BCG. Figure S2: The non-programmed cell death (non-PCD) symptoms of BCG and PhCAO were alleviated by overexpressing HCAR.
