# Supplementary material for: HCAR Is a Limitation Factor for Chlorophyll Cycle and Chlorophyll b Degradation in Chlorophyll-b-Overproducing Plants
Source: Biomolecules. 2020 Dec 5;10(12):1639. doi: 10.3390/biom10121639 (PMC7762049; doi:10.3390/biom10121639)
Supplement: Supplementary file 1 [file biomolecules-10-01639-s001.pdf]

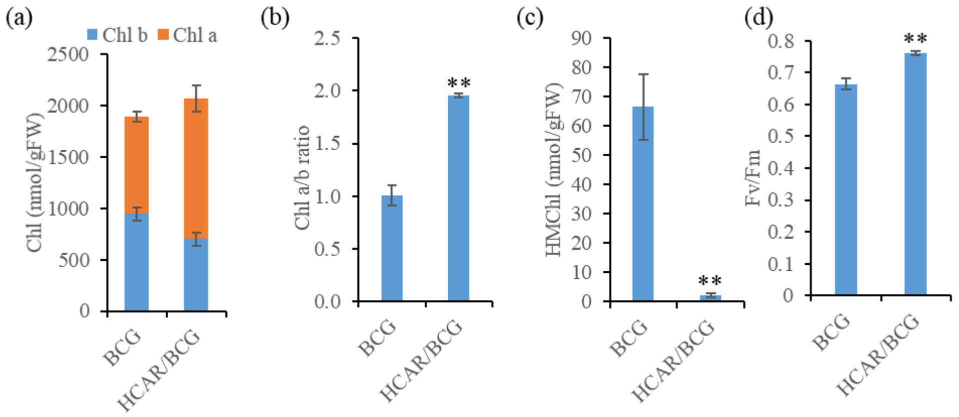

Figure S1. The changes of Chl and Chl fluorescence parameters upon overexpressing HCAR in BCG. (a) Chl *a* and *b* contents, (b) Chl *a*/*b* ratios, (c) HMChl *a* contents and (d) Fv/Fm ratios of BCG and HCAR/BCG plants. Values represent the mean of three replicate samples ± SD. Asterisks indicate significant difference compared to BCG (Student's t-test, ** $p < 0.01$).

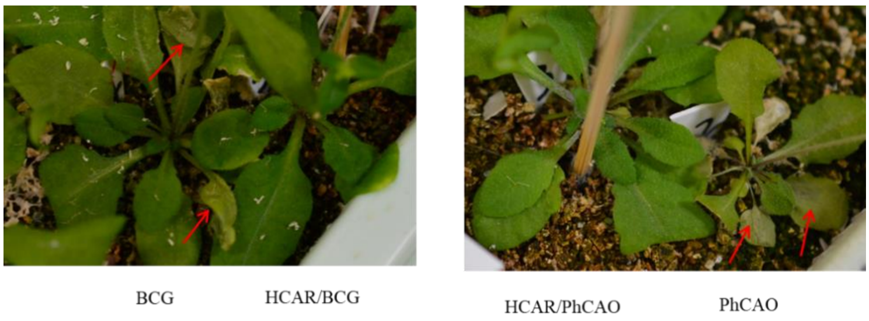

Figure S2. The non- programmed cell death (non-PCD) symptoms of BCG and PhCAO were alleviated by overexpressing HCAR. Plants were grown under long-day conditions for 7 weeks. Red arrows indicate the non-PCD symptoms of old leaves.
